# A Transcription Factor *Sl*NAC4 Gene of *Suaeda liaotungensis* Enhances Salt and Drought Tolerance through Regulating ABA Synthesis

**DOI:** 10.3390/plants12162951

**Published:** 2023-08-15

**Authors:** Jiahui Liu, Hongfei Wang, Mingxing Su, Qianqian Li, Honglin Xu, Jieqiong Song, Changping Li, Qiuli Li

**Affiliations:** Key Laboratory of Plant Biotechnology of Liaoning Province, School of Life Sciences, Liaoning Normal University, Dalian 116081, China

**Keywords:** *Suaeda liaotungensis* K., NAC transcription factor, abiotic stress, transgenic *Arabidopsis thaliana*, abscisic acid

## Abstract

The NAC (NAM, ATAF1/2 and CUC2) transcription factors are ubiquitously distributed in plants and play critical roles in the construction of plant organs and abiotic stress response. In this study, we described the cloning of a *Suaeda liaotungensis* K. NAC transcription factor gene *SlNAC4*, which contained 1450 bp, coding a 331 amino acid. We found that *SlNAC4* was highly expressed in stems of *S. liaotungensis*, and the expression of *SlNAC4* was considerably up-regulated after salt, drought, and ABA treatments. Transcription analysis and subcellular localization demonstrated that the *Sl*NAC4 protein was located both in the nucleus and cytoplasm, and contained a C-terminal transcriptional activator. The *SlNAC4* overexpression *Arabidopsis* lines significantly enhanced the tolerance to salt and drought treatment and displayed obviously increased activity of antioxidant enzymes under salt and drought stress. Additionally, transgenic plants overexpressing *SlNAC4* had a significantly higher level of physiological indices. Interestingly, *Sl*NAC4 promoted the expression of ABA metabolism-related genes including *AtABA1*, *AtABA3*, *AtNCED3*, *AtAAO3*, but inhibited the expression of *AtCYP707A3* in overexpression lines. Using a yeast one-hybrid (Y1H) assay, we identified that the *Sl*NAC4 transcription factor could bind to the promoters of those ABA metabolism-related genes. These results indicate that overexpression of *SlNAC4* in plants enhances the tolerance to salt and drought stress by regulating ABA metabolism.

## 1. Introduction

Multiple abiotic stresses such as high salinity, drought, and low temperature cause osmotic stress, ion toxicity and oxidative damage in plants, and have negative effects on plant growth and development. In order to survive smooth under adverse environmental conditions, plants have evolved various defense mechanisms involving changes at the cellular, molecular, physiological, and biochemical levels [1]. Transcription factors (TFs) are the key factors mediating the response to abiotic stress signals to precisely regulate the expression of downstream genes [2]. The central function of TFs is their ability to bind specific DNA sequences and interact with different proteins in transcriptional complexes that regulate the expression of a vast number of genes [3]. Many TF families, such as NAC, WRKY, DREB, MYB, bZIP are known to be involved in the response mechanisms of abiotic stresses in plants [4,5,6]. As one of the largest plant-specific TF families, the NAC (NAM, CUC, and ATAF) TF family usually comprises a quite conserved NAC domain and an extremely variable transcription regulatory region (TRR) [7]. The NAC domain is situated at the N-terminal and contains nearly 150 amino acid residues, which can be divided into five major subdomains (named A to E) [8]. TRR is situated at the C-terminal and can activate or repress gene transcription [9].

In recent years, many studies have demonstrated that NAC TFs play indispensable roles in multiple abiotic stresses. For example, overexpression of *AtJUB1*(a NAC TF gene) from *Arabidopsis thaliana* enhances drought tolerance by increasing the relative leaf water content in tomato [10]; and as a positive regulator of drought tolerance, *At*HB1(a HD-ZipI TF) and JUB1 establish a joint drought stress control module [11]. As a mediator of drought stress, *Pw*NAC11 activates ERD1 by interacting with ABF3 and DREB2A to positively respond to early drought stress in transgenic *Arabidopsis* [12]. *TaNAC29* is a gene cloned from bread wheat that has high homology to *NAC29* from *Aegilops tauschii. Ta*NAC29 is involved in the regulation of *SAG113* expression to enhance drought and salt stresses [13]. Rice plants overexpressing *OsNAC5*, *6*, *9*, and *10* have greater drought resistance and reduced grain yield loss as compared with the wild-type plants. As the cellular components, *Os*NACs alter the root architectures of *RCc3:6MYC-OsNACs* rice lines for drought tolerance [14]. Besides, *Os*NAC3 is involved in ABA response and salt tolerance in rice through regulating the expression of stress-responsive genes and shoot Na^+^ homeostasis [15]. The *Ca*NAC46 transcription factor gene was identified from *Capsicum annuum*, which belongs to the ATAF subfamily. Induced by drought and salt stresses, *Ca*NAC46 activated ROS-scavenging enzymes and enhanced root formation [16]. *ThNAC12-overexpressing* transgenic plants increased salt tolerance by regulating ROS-scavenging capability and antioxidant enzyme activity, and *ThNAC4* overexpression in *Arabidopsis* plants regulates the synthesis of osmoregulators, thereby enhancing salt tolerance and drought resistance in transgenic plants [17,18]. Overexpression of the *GmNAC06* gene from soybean (*Glycine max* L. Merr) maintained ionic homeostasis and osmotic equilibrium and increased salt tolerance in both the germination and seeding stages [19]. *LpNAC13* attenuated drought tolerance but increased the tolerance of tobacco plants to long-term salt stress [20]. *NtNAC053* gene was also isolated in tobacco, which could enhance the salt and drought tolerance through inspiring the downstream stress-responsive genes and antioxidant system [21]. *AvNAC030* is a NAC gene that was screened from kiwifruit. Compared with the control, the electrolyte leakage and malnodialehyde value were lower in *AvNAC030*-overexpressing *Arabidopsis*. In addition, the overexpression plants had improved antioxidant defense mechanisms. So that *AvNAC030* can increase the salt tolerance of plants [22]. *MdNAC25*-overexpressing *Arabidopsis* plants improved the tolerance to cold and salinity stress via the enhanced scavenging capability of reactive oxygen species (ROS) [23]. The cold-responsive *MaNAC1* that is induced by ethylene may be involved in the cold tolerance of banana fruits through its interaction with ICE1-CBF cold signaling [24]. However, *MaNAC25* and *MaNAC28* negatively regulated cold tolerance through up-regulating the expression of phospholipid degradation genes in banana fruits [25]. Overexpressing *HuNAC20* and *HuNAC25* in *Arabidopsis* increased tolerance of cold stress by altering the expression of cold-expression genes in transgenic plants [26].

*Suaeda liaotungensis* K., an annual herb of Amaranthaceae, is a typical halophyte growing in arid or semi-arid saline-alkali soil. *S. liaotungensis* has complex mechanisms for response to abiotic stresses, which is a good material for studying the mechanism of salinity and drought tolerance in plants. Based on its superior properties, it is extremely important to screen and reveal the cellular and molecular mechanisms of its abiotic resistance. In previous studies, we conducted transcriptome analysis of *S. liaotungensis* after salt stress and found several highly expressed NAC transcription factor genes. So far, we have reported five NAC transcription factor genes (*SlNAC1*, *SlNAC2*, *SlNAC7*, *SlNAC8*, and *SlNAC10*) from *S. liaotungensis*, which changed the physiological-biochemical characteristics of plants by regulating downstream stress response genes, and enhanced plant tolerance to abiotic stress [27,28,29,30,31]. In this study, based on transcriptomic data, we isolated another NAC transcription factor gene named *SlNAC4* from *S. liaotungensis*. Sequence analysis indicated that the *SlNAC4* cDNA fragment is 1450 bp long, including an open reading frame of 996 bp, which encodes 331 amino acids. We found that the *Sl*NAC4 protein showed both nuclear and cytoplasmic localization and had activity of a transcriptional activator. The *SlNAC4*-overexpression lines showed significant advantages in physiology and morphology after salt and drought stress compared to the wild-type and demonstrated enhanced resistance to salt and drought stress. Additionally, *Sl*NAC4 could bind to the promoters of ABA metabolism-related genes, such as *Arabidopsis ABA1* (*AtABA1*, a zeaxanthin epoxidase gene that functions in the first step of the biosynthesis of the abiotic stress hormone abscisic acid), *Arabidopsis ABA3* (*AtABA3*, a molybdenum cofactor sulfurase gene that is involved in the last step of abscisic acid biosynthesis), *Arabidopsis NCED3* (*AtNCED3*, encoding a key enzyme named nine-cis-epoxycarotenoid dioxygenase 3 in the biosynthesis of abscisic acid), *Arabidopsis AAO3* (*AtAAO3*, an abscisic aldehyde oxidase 3 gene that catalyzes the final step in abscisic acid biosynthesis), and *Arabidopsis CYP707A3* (*AtCYP707A3*, encoding a protein with ABA 8’-hydroxylase activity, which is involved in ABA catabolism), and regulate the transcription of their downstream genes. The ultimate goal is to improve the ability of plants to cope with abiotic stresses by regulating ABA content. Overall, these findings reveal the stress resistance mechanism of *SlNAC4* mediated by regulating ABA synthesis, which helps us understand the vital function of the *SlNAC4* transcription factor in improving the resistance to salt and drought stress in transgenic *Arabidopsis*, and provide a more theoretical basis for the cultivation of crop varieties.

## 2. Results

### 2.1. SlNAC4 Encodes a Protein Containing the NAC Domain

**Figure 1:**
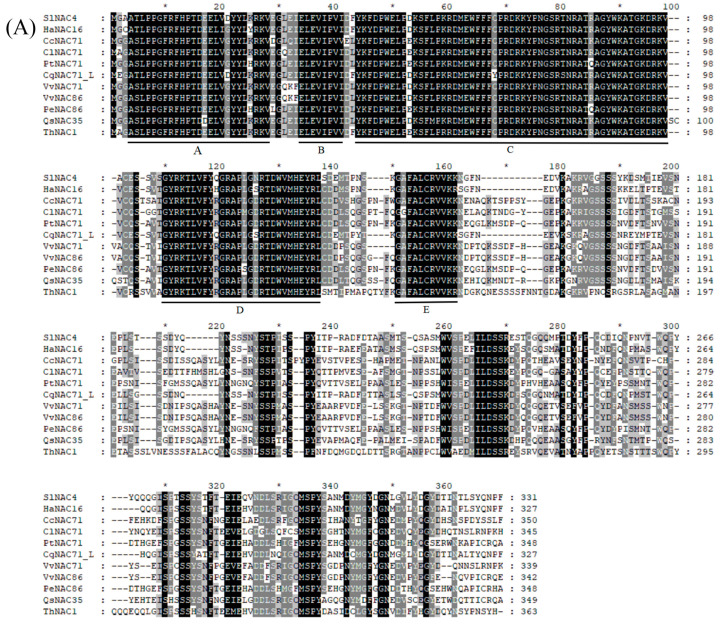

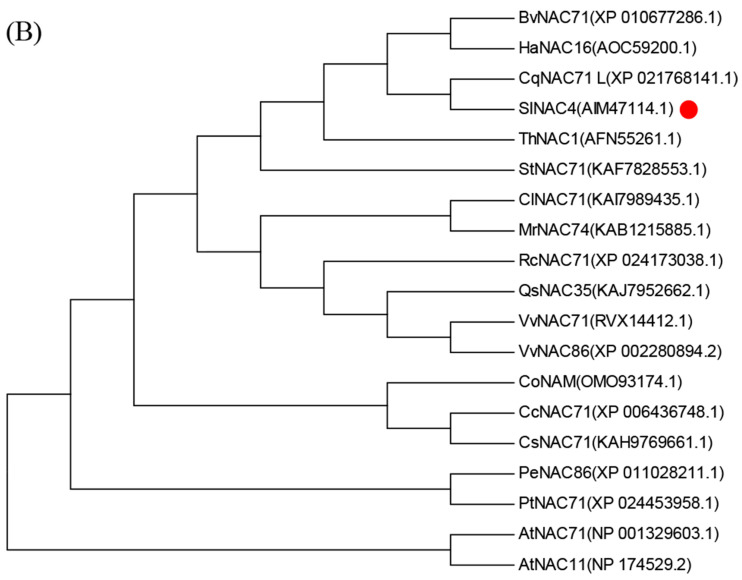
Multiple sequence alignment and phylogenetic analysis of *Sl*NAC4 with homologous NAC protein. (**A**) Protein multiple sequence alignment of *Sl*NAC4. The five subdomains of the NAC domain are denoted by lines designated with the letters A–E. (**B**) Phylogenetic analysis of *Sl*NAC4 and other plant NAC protein. The dendrogram was constructed using MEGA 5.0 software. The asterisks indicate positions which have a single, fully conserved residue. Numbers on each branch represent the confidence of 1000 repetitions. *Sl*NAC4 is labeled with a large red dot.

The length of the *SlNAC4* cDNA fragment amplified by PCR was 1450 bp, and contains an ORF of 996 bp, which encodes 331 amino acids. Conserved domain program analysis and multiple sequence alignment showed that the *Sl*NAC4 protein had a NAM domain comprising five subdomains (A, B, C, D, and E) (Figure 1A). Phylogenetic analysis indicated that *Sl*NAC4 has the highest similarity to *Cq*NAC71_L, *Ha*NAC16, *Bv*NAC71, and *Th*NAC1 (the members of the NAM subfamily), indicating that *Sl*NAC4 belongs to the NAM subfamily (Figure 1B).

### 2.2. SlNAC4 Is Responsive to Various Abiotic Stresses in Suaeda liaotungensis

**Figure 2:**
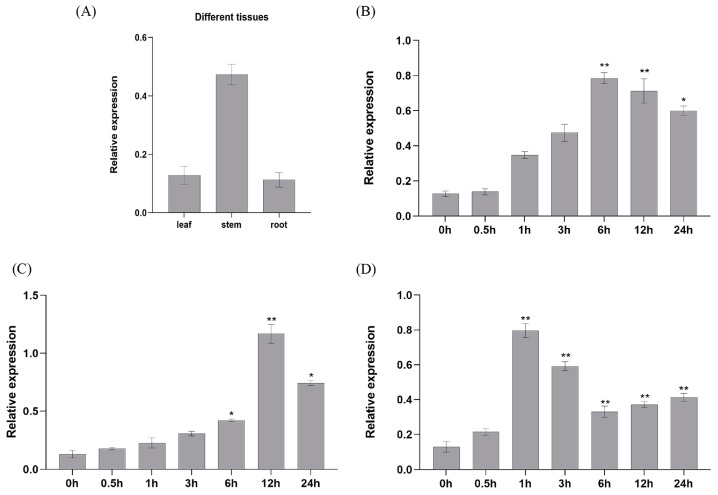
Expression patterns of *SlNAC4* in S. liaotungensis. (**A**) *SlNAC4* expression in the leaf, stem, and root under normal conditions. Expression pattern of *SlNAC4* after exposure to (**B**) 200 mM NaCl, (**C**) 20% polyethylene glycol, and (**D**) 0.1 mM abscisic acid treatment. The error bars represent ± SD, and the asterisks indicate significant differences (* *p* < 0.05, ** *p* < 0.01).

In the present study, the expression level of *SlNAC4* in different organs of *Suaeda liaotungensis* and in response to different stresses and ABA stimulation were investigated with qRT-PCR. The results showed that the expression of *SlNAC4* was the highest in stems and nearly fivefold that in leaves and roots (Figure 2A), indicating that *SlNAC4* may play a vital role in the vegetative growth of plants. Furthermore, we analyzed the expression pattern of *SlNAC4* in leaves under NaCl, polyethylene glycol, and exogenous ABA treatment. After 200 mM NaCl and 20% polyethylene glycol (PEG) treatments, *SlNAC4* expression was dramatically induced after 6 h (Figure 2B,C). Interestingly, in response to treatment with the hormone ABA, we found that the *SlNAC4* can be significantly induced by ABA and that its expression reached a maximum after 1 h of treatment (Figure 2D). These results indicate that *SlNAC4* has different expression in organs, and may participate in the growth procession of *Suaeda liaotungensis*. Abiotic stress conditions (salt and drought) and hormonal (ABA) stimulation also can induce *SlNAC4* transcription.

### 2.3. SlNAC4 Is Located in Both Nucleus and Cytoplasm

**Figure 3:**
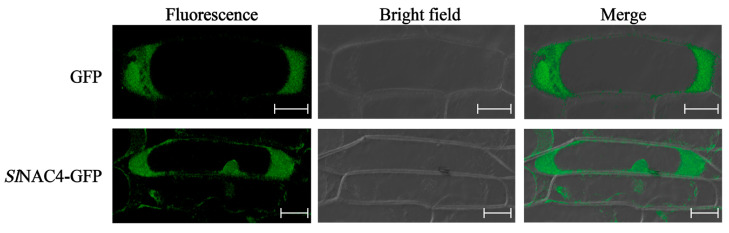
Subcellular localization of *Sl*NAC4 fused with GFP. Onion epidermal cells co-expressing GFP or *Sl*NAC4-GFP under the control of CaMV35S promoter. Fluorescence: green fluorescence image. Merge: the merged images of the bright field and fluorescence. Scale bar = 50 µm.

To investigate the subcellular localization of *Sl*NAC4 in living cells, the fusion vector *Sl*NAC4-GFP and GFP empty vector were transformed into onion epidermal cells using the gene gun method for transient expression. GFP fluorescence of the GFP vector was dispersed throughout the whole cell, and we also observed GFP fluorescence of *Sl*NAC4-GFP in the nucleus and cytoplasm of cells (Figure 3). This result determines that *Sl*NAC4 shows both nuclear and cytoplasmic localization.

### 2.4. SlNAC4 Exhibits Transcriptional Activation Activity

**Figure 4:**
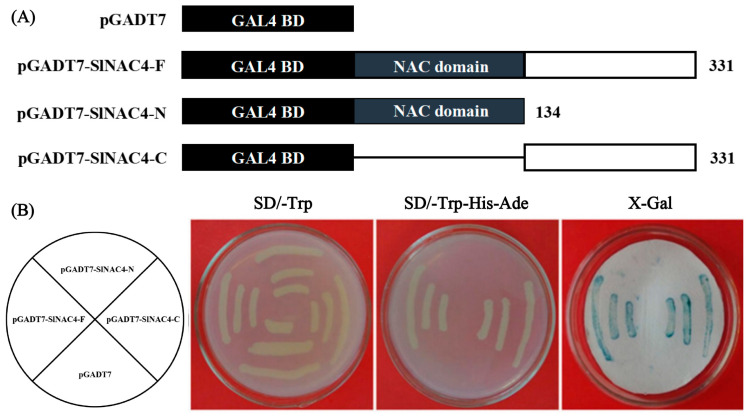
Transactivation assay of *Sl*NAC4 in yeast. (**A**) Schematic diagrams of the pGBKT7 vector, full-length (pGBKT7-*SlNAC4*-F), N-terminus (pGBKT7-*SlNAC4*-N), and C-terminus (pGBKT7-*SlNAC4*-C) of *Sl*NAC4 were fused with GAL4 BD. The numbers indicate the positions of the amino acids. (**B**) Growth on the selective medium of yeast cells transformed with different constructs using pGBKT7 as a control. X-Gal medium was used to verify β-galactosidase activity.

To investigate the transcriptional activation activity of *Sl*NAC4, we respectively fused the full-length coding sequence, N-terminal domain, and C-terminal domain of *SlNAC4* with the GAL4 DNA binding domain in the pGBKT7 vector (Figure 4A), which was then transferred into yeast strain AH109. The empty pGBKT7 vector was used as a negative control. The results showed that all yeast cells grew well on SD/-Trp medium. However, only yeast cells containing pGBKT7-*SlNAC4*-F and pGBKT7-*SlNAC4*-C could grow on SD/-Trp/-His/-Ade medium, and turn blue on X-Gal medium (Figure 4B), indicating that *Sl*NAC4 is a transcriptional activator with an activation region in its C-terminus.

### 2.5. Overexpression of SlNAC4 Increases the Salt and Drought Tolerance of Transgenic Arabidopsis thaliana

**Figure 5:**
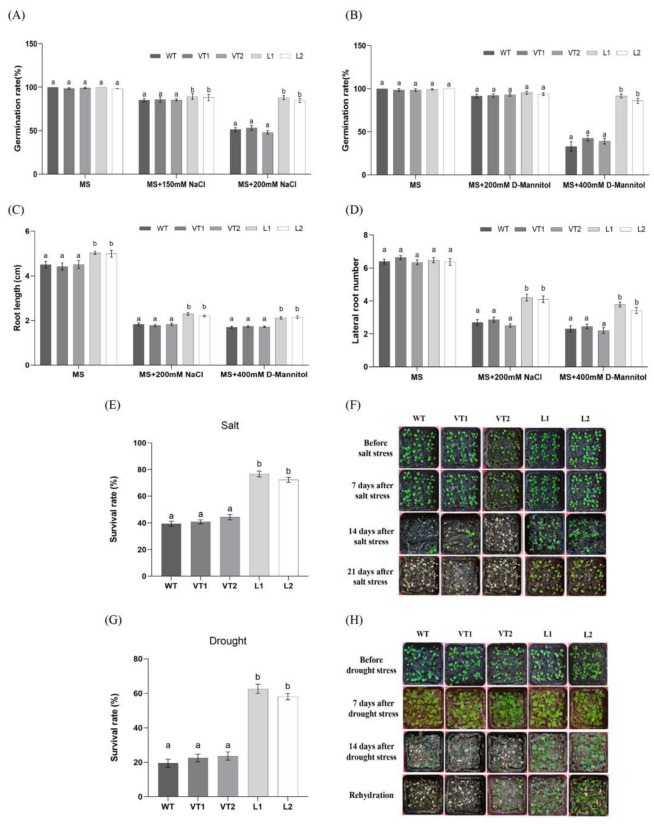
The growth and survival rate of wild-type (WT), empty vector lines (VT1, VT2), and *SlNAC4*-overexpression *Arabidopsis* lines (L1, L2). Germination rates of wild and transgenic lines under (**A**) MS, MS +150 mM, and MS + 200 mM NaCl, (**B**) MS, MS + 200 mM, and MS + 400 mM D-mannitol. (**C**) The primary root length and (**D**) lateral root number of wild and transgenic lines under MS, MS + 200 mM, NaCl and MS + 400 mM D-mannitol. Survival percentage of wild and transgenic lines under (**E**) salt stress, (**G**) drought stress. Phenotypes of different lines at (**F**) salt treatment, (**H**) drought treatment. Each bar represents the mean ± SD of triplicate experiments (different lowercase letters indicate significant differences between the different lines, *p* < 0.05).

In order to investigate the role of *SlNAC4* under abiotic stress, two transgenic *Arabidopsis* lines overexpressing *SlNAC4* were obtained. After salt and drought stress treatment, seedlings of wild-type (WT), empty vector lines (VT1, VT2), and overexpressing lines (L1, L2) showed different phenotype (Supplementary Figure S1). After three days of culture on common MS medium, the seeds of all lines germinated normally, indicating that those seeds had well-developed embryo structure and germination ability. Under the salt and drought stress, the germination rate of WT and VT lines were more affected than that of overexpression lines. We found that under normal conditions, in the presence of 150 mM NaCl and 200 mM D-mannitol, there is no significant difference between controls and *SlNAC4*-transgenic plants. However, the germination percentage of L1 and L2 was over 80% under 200 mM NaCl conditions compared to less than 60% of the WT and VT lines. Moreover, under the treatment of 400 mM D-mannitol, the germination rate of overexpression lines was higher than WT and VT lines, showing a significant difference (Figure 5A,B). These results indicate that L1 and L2 have stronger seed vitality in the early stage of germination, which suggested that the transfer of the *SlNAC4* gene enhanced the germination ability of *Arabidopsis*.

In the root elongation and lateral root growth experiment, we found that the roots of *SlNAC4* overexpression lines were longer than those of WT and VT lines under normal culture conditions. Fifteen days after the 200 mM NaCl and 400 mM D-mannitol treatments, though the root growth of all lines was inhibited, the root length of overexpression plants was still notably higher than that of WT, VT1, and VT2 (Figure 5C). The lateral root growth produced similar results; *SlNAC4* transgenic *Arabidopsis* have more lateral roots (Figure 5D).

To examine the effect of *SlNAC4* on plant stress resistance, three-week-old seedlings with the same growth status were selected for stress treatment. Under salt stress (200 mM NaCl), we observed that the survival ratio of L1 and L2 were 75% and 72%, respectively, which were significantly higher than those of the WT (39%), VT1 (40.3%), and VT2 (44.6%) lines (Figure 5E,F). In addition, the overexpression lines only showed leaf shrinkage and still maintained a normal growth state with a final survival ratio of 62.5% and 58.7% after drought stress (rehydrating for 3 days after 21 days without watering) and following by re-watering. In contrast, the leaves of the control groups withered or even fell off, or even died, and were unable to return to normal after re-watering, and the final survival rate was only 19%, 21%, and 24% (Figure 5G,H).

### 2.6. Overexpression of SlNAC4 Enhances Physiological Indices in Transgenic Arabidopsis thaliana

**Figure 6:**
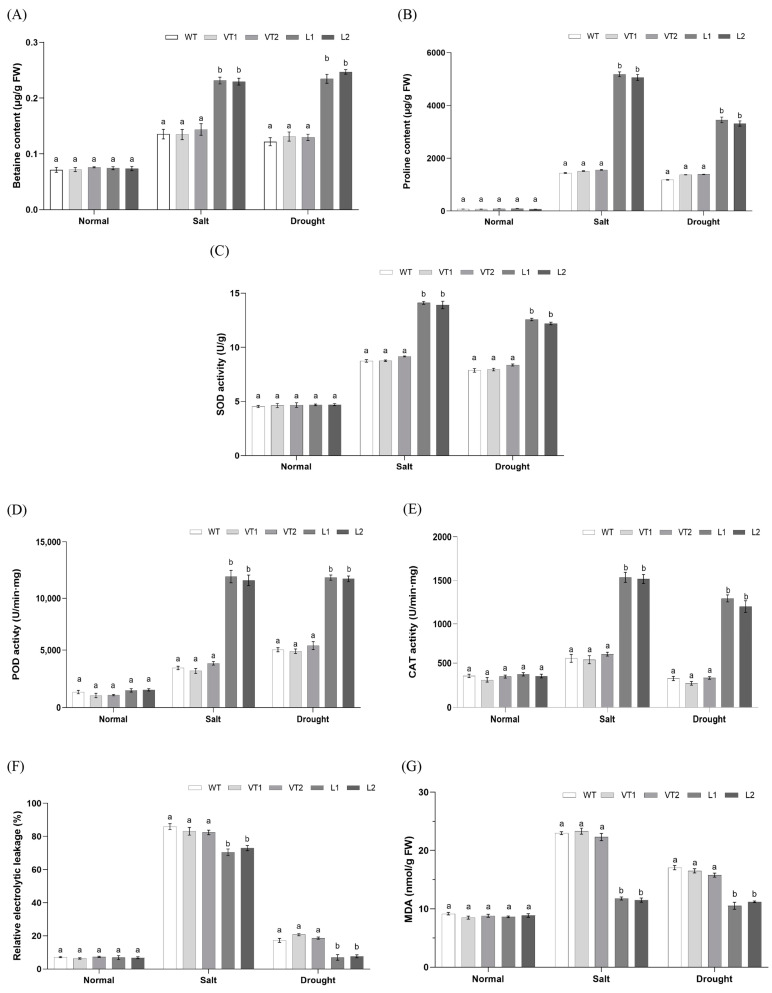
Physiological analyses of wild-type (WT), empty vector lines (VT1, VT2), and *SlNAC4*-overexpression *Arabidopsis* lines (L1, L2) under salt and drought stress. (**A**) the content of betaine; (**B**) the content of proline; (**C**) SOD activity; (**D**) POD activity; (**E**) CAT activity; (**F**) the relative electrical conductivity; (**G**) the concentration of MDA. Each bar represents the mean ± SD of triplicate experiments. Different letters indicate significant differences at *p*-value < 0.05.

Both proline and betaine are important osmoregulatory substances, which can participate in the osmoregulation of plant cells and play a vital role in maintaining plant metabolism under abiotic stress conditions. The analysis of the contents of proline, betaine revealed no significant difference between controls and *SlNAC4*-transgenic plants under normal conditions. On the contrary, overexpression lines resulted in higher proline and betaine levels than corresponding control plants under salt and drought stress (Figure 6A,B). This indicated that overexpression of *SlNAC4* may better maintain osmotic balance and protect cellular structures from damage caused by salt and drought stress.

When the content of ROS in the plant increases, it will cause damage to the membrane system and protein, and pathological changes in the body. Antioxidant enzymes such as superoxide dismutase (SOD), peroxidase (POD), and catalase (CAT) can remove ROS and protect the normal life activities of the plant. Our analyses of the antioxidant enzyme activities revealed no appreciable differences between transgenic plants and control plants under normal conditions. However, the activities of SOD, POD, and CAT in *SlNAC4* overexpression plants were apparently higher than those in WT, VT1, and VT2 during abiotic stress (Figure 6C–E). Furthermore, the severity of cytomembrane oxidative damage was reflected in the MAD content. Under normal conditions, we found there was no significant difference in MDA concentration between *SlNAC4* transgenic lines and non-transgenic lines. When subjected to salt and drought stress, significantly lower MDA levels were detected in *SlNAC4*-overexpressing plants than WT, VT1, and VT2 lines (Figure 6G). The same results showed in the conductivity assay (Figure 6F), indicating that the degree of cell membrane damage of *SlNAC4* transgenic plants was lighter than that of non-transgenic plants. This study reported that overexpressing *SlNAC4* probably enhances the antioxidant activity under abiotic stress, improves the scavenging capability of ROS, and helps plants avoid peroxidation damage.

**Figure 7:**
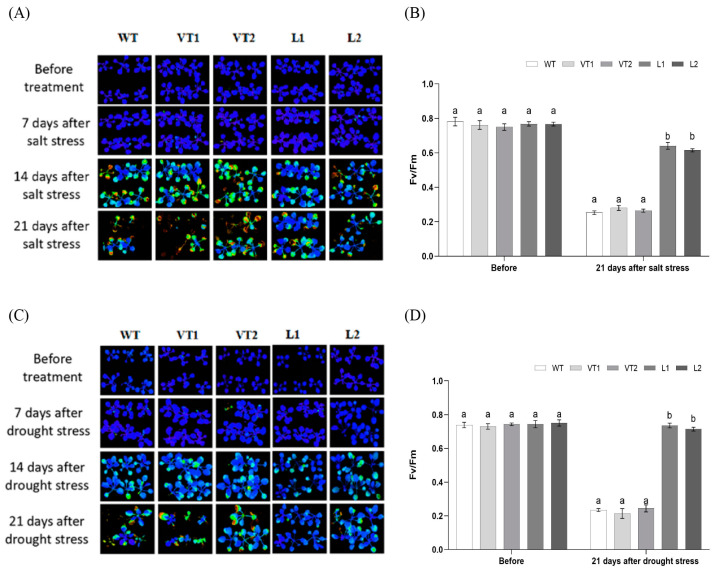
Chlorophyll fluorescence analysis of wild-type (WT), empty vector lines (VT1, VT2), and *SlNAC4*-overexpression *Arabidopsis* lines (L1, L2) under salt and drought stress. The chlorophyll florescence and photosynthetic efficiency (Fv/Fm) of wild and transgenic *Arabidopsis* under (**A**,**B**) salt treatment, (**C**,**D**) drought treatment. Each bar represents the mean ± SD of triplicate experiments. Different letters indicate significant differences at *p*-value < 0.05.

Several studies have evaluated the deleterious effects of high salt and drought on photosynthesis. Drought disrupts photosynthetic pigments and reduces water-use efficiency; meanwhile, salt can cause irreversible damage to photosynthetic apparatus at any developmental stage. Chlorophyll fluorescence analysis is an intuitive and effective technique to measure the photochemical efficiency of PSII and observe the degree of plant damage. The present study measured photosynthetic efficiency (Fv/Fm) to verify the function of *SlNAC4* in photosynthesis under abiotic stress. The Fv/Fm of each line was between 0.7 and 0.8 before treatment, indicating that the growth status of each *Arabidopsis* line was normal. After 21 days of salt stress and drought stress, the chlorophyll fluorescence of different lines was weakened to varying degrees, and the color changed from blue to red. Under salt stress, the Fv/Fm value of L1 and L2 showed a slight drop to 0.65 and 0.62. While the value of WT, VT1, and VT2 plants were only between 0.21 and 0.24 (Figure 7B). Under drought stress, the Fv/Fm value of L1 and L2 plants were 0.76 and 0.75, compared with the control group values of 0.2, 0.21, and 0.22, respectively (Figure 7D). This result suggested that the transfer of *SlNAC4* increased the photoprotection in L1 and L2 lines.

### 2.7. Overexpression of SlNAC4 Activates the Expression of ABA Metabolism Related Genes and ABA Sythesis

**Figure 8:**
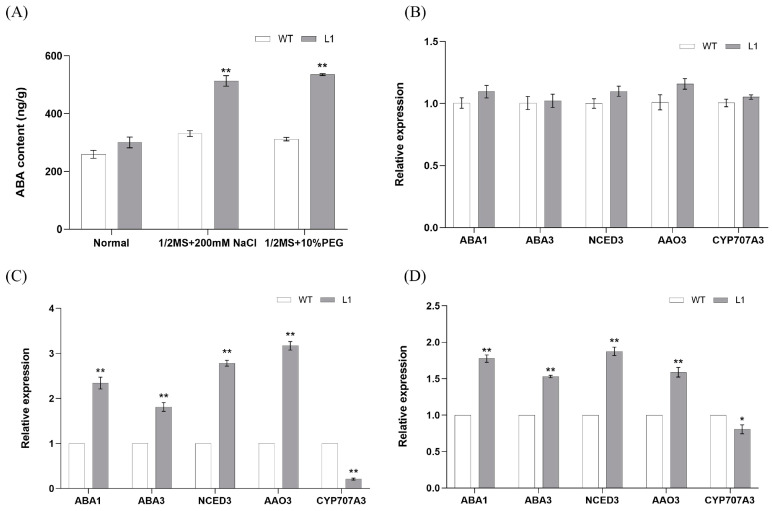
The ABA content and expression of five ABA-metabolism genes in *SlNAC4* transgenic line (L1) and wild-type *Arabidopsis* (WT) under different abiotic stress. (**A**) the content of ABA; (**B**) under normal condition; (**C**) under 200 mM NaCl treatment; (**D**) under 10% PEG treatment. The asterisks above the columns indicate a significant difference compared to WT (* *p* < 0.05, ** *p* < 0.01).

ABA functions as a messenger in biological processes such as seed dormancy and germination, leaf senescence, and stress response. Once subjected to biotic or abiotic stress, the ABA content increases significantly, activating the corresponding receptors to initiate signal transduction in response to stress. Our study found that the content of ABA increased significantly in *SlNAC4* transgenic *Arabidopsis thaliana* under 200 mM NaCl and 10% PEG treatment (Figure 8A).

Given the results we observed using transgenic plants, ABA was increased in *SlNAC4* overexpressing plants, accompanied by enhanced stress resistance. To further explore the regulating role of *Sl*NAC4 on ABA metabolism, we analyzed the expression of five ABA metabolism-related genes (*AtABA1*, *AtABA3*, *AtNCED3*, *AtAAO3*, and *AtCYP707A3*) in the WT and overexpression *Arabidopsis* line (L1) using qRT-PCR. The results showed that there were no significant differences in the expression of the above five genes between WT and L1 *Arabidopsis* plants under control conditions. Under the stimulation of salt and drought, the expression of *AtABA1*, *AtABA3*, *AtNCED3*, *AtAAO3* in the overexpressing-*SlNAC4* line was substantially up-regulated compared with WT; by contrast, *AtCYP707A3* had the opposite performance (Figure 8B–D). The finding suggested that *SlNAC4* might enhance salt and drought tolerance by regulating the expression of ABA metabolism-related genes.

### 2.8. SlNAC4 Protein Binds to the Promoters of AtABA1, AtABA3, AtNCED3, AtAAO3 and AtCYP707A3

**Figure 9:**
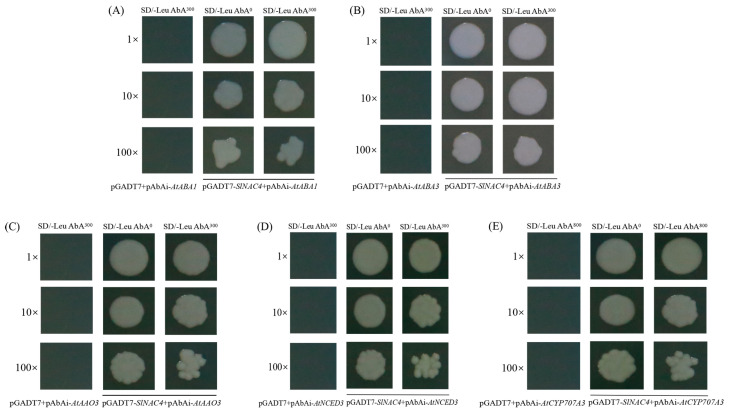
Identification of interactions between *Sl*NAC4 and ABA metabolism-related genes. Yeast one-hybrid assays showed that *Sl*NAC4 transcription factor binds to the promoters of (**A**) *AtABA1*, (**B**) *AtABA3*, (**C**) *AtAAO3*, (**D**)*AtNCED3*, (**E**) *AtCYP707A3*.

We found that several -CGT(G/A) and -CACG motifs were harbored in the promoter regions of *AtABA1*, *AtABA3*, *AtNCED3*, *AtAAO3*, and *AtCYP707A3*; these motifs, named NAC recognition sequence (NACRS), were identified as the core conserved binding sites of NACs. To verify the protein-DNA interactions, the yeast one-hybrid assay was applied to detect whether the *Sl*NAC4 transcription factor can interact with the promoter of *AtABA1*, *AtABA3*, *AtNCED3*, *AtAAO3*, and *AtCYP707A3*. Based on the results, the final Aureobasidin A (AbA) concentration of 300 ng/mL and 800 ng/mL were determined as the background expression level for the bait yeast. The bait yeast transformed with empty pGADT7 was used as the control to observe the growth of yeast transformed with pGADT7-*SlNAC4* recombinant vector. The recombinant yeast cells grew well on SD/-Leu^0^, SD/-Leu ABA^300^ and SD/-Leu ABA^800^ medium (Figure 9), indicating that the *Sl*NAC4 transcription factor can bind to *AtABA1*, *AtABA3*, *AtNCED3*, *AtAAO3*, and *AtCYP707A3* promoters (Figure 9). It is speculated that *Sl*NAC4 can directly regulate the expression of ABA metabolism-related genes, so as to increase the content of ABA and enhance the ability of plants to resist abiotic stress.

## 3. Discussion

NAC is one of the largest plant-specific transcription factor families and has crucial functions in the regulation of defense against harsh environmental stresses [32]. Numerous NAC transcription factors from various plants have been shown to operate in ABA-dependent and -independent signaling pathways [33]. *ZmNAC55* and *ZmNAC84* from maize [34,35], *OsNAC2* and *OsNAP* from rice [36,37], *CaNAC19*, *CaNAC41*, and *CaNAC57* from chickpea [38], and *GhirNAC2*, *GhNAC4*, and *GhATAF1* from cotton [39,40,41] play vital roles in the response to abiotic stress by coordinating the ABA signaling network. However, little information is available about functional identification of the NAC family in *Suaeda liaotungensis.* In this study, we demonstrated that NAC TF *Sl*NAC4 could coordinate the physiological and molecular responses of transgenic *Arabidopsis* to abiotic stress by directly regulating the expression of ABA metabolism-related genes.

In the present study, we cloned a novel NAC TF gene named *SlNAC4* from *Suaeda liaotungensis*. The multiple sequence alignments revealed that *Sl*NAC4 had a conserved NAC structural domain containing five subdomains at the N-terminal region, and a variable domain at the C-terminal region. To explore the phylogenetic relationship of *Sl*NAC4, a phylogenetic tree was constructed. Phylogenetic analysis showed that *Sl*NAC4 fell into the NAM subfamily and had the closest relationship with *Cq*NAC71_L, *Ha*NAC16, *Bv*NAC71, and *Th*NAC1 (Figure 1). The NAM subfamily transcription factors play vital roles in abiotic stress resistance. For example, overexpression of *TaNAC2* (a wheat NAC gene) could improve drought tolerance in transgenic tobacco plants [42]. It has been reported that the expression of the NAM subfamily member *At*NAC2 can be induced by salt stress [43]. Thus, we hypothesized that *Sl*NAC4 may also play an important role in the response to abiotic stress.

To explore the potential function of *SlNAC4*, the expression pattern analysis was carried out. We found that *SlNAC4* could be strongly induced by salt, drought, and ABA stress (Figure 2). Furthermore, *Sl*NAC4 could be realized in the nucleus and cytoplasm (Figure 3), which was extremely different with most NAC transcription factors [44]. When plants are subjected to multiple abiotic stresses, the first obstacle encountered is the energy crisis. However, a mechanism has been proposed to deal with this dilemma by reducing polyribosome (polysome) complex formation, or loading multiple ribosomes on single mRNA [45]. Early molecular studies of plant responses to hypoxia and high temperature recognized that stress up-regulated gene transcripts were prioritized over many other polyadenylated mRNAs [46,47]. Therefore, we speculated that *Sl*NAC4 present in the cytoplasm could improve the translation efficiency and regulate the expression of stress-related genes in time to ensure the normal growth state of plants. Overexpression of *ThNAC4* improved physiological parameters such as seed germination rate, root length, and fresh weight of transgenic *Arabidopsis* [18]. Various NAC proteins have been reported to have a transcription activation functional region, such as *Zm*NAC074, *Bn*NAC2, and *Ml*NAC12 [48,49,50]. Transcription activation assays showed that *Sl*NAC4 possessed transcriptional activation activity and the transcriptional activation domain is located in the C-terminal region (Figure 4).

In previous studies, we have shown that *Sl*NAC TFs belong to different subfamilies and can regulate the expression of downstream stress-related genes, thereby altering the physiological-biochemical characteristics of plants under adverse conditions and enhancing the stress tolerance of transgenic plants [27,28,29,30,31]. Based on the above findings, we hypothesized that *Sl*NAC4 also has a similar function, thus, we overexpressed *SlNAC4* in *Arabidopsis* to validate our hypothesis and better analyze the molecular function of *SlNAC4*. Environmental conditions have been reported to play important roles in seed germination [51]. Meanwhile, the root system is highly sensitive to changes in its surrounding environment and abiotic stress such as salinity, and drought can significantly affect the root length and lateral root number of a plant [52]. In the current study, we found that the seed germination rate, root length, and lateral root number of overexpression lines under salt and drought conditions were evidently higher than those of WT plants (Figure 5A–D). This finding is similar to the report that *PgNAC21*-overexpressing *Arabidopsis* plants improves adaption to adversity by altering root architecture [53]. The *GhNAC3* gene conferred drought tolerance to *Arabidopsis* as shown by enhanced seedling survival and the cotyledon rate [54]. Similarly, in this study, *SlNAC4*-harboring *Arabidopsis* previously showed a higher survival rate compared to control plants when exposed to salt and drought stress (Figure 5E–H). Considering these results, the overexpression of *SlNAC4* gene could enhance the tolerance to salt and drought stresses in transgenic *Arabidopsis*.

High-salt and drought-induced ion stress causes the accumulation of excessive reactive oxygen species in plants, which damages membrane lipids, proteins, and nucleic acids [55]. Therefore, the content of several osmoprotectants tend to increase under stress conditions. Numerous various studies have shown that exogenous proline can improve the growth, yield, and stress tolerance in plants under adverse environmental factors [56]. Meanwhile, several studies have established the crucial roles of glycine betaine (GB) as a compatible osmolyte in plants for enhancing plant tolerance to abiotic stresses, including salinity, drought, and low temperature [57,58,59]. Additionally, exogenous application of GB could protect the photosystem II complex (PSII) through maintaining PSII’s stability under various abiotic stresses [60]. Besides osmotic adjustment substances, there are several antioxidant enzymes-POD, SOD, and CAT-acting as a protective enzyme system to limit the levels of free radicals and prevent their damage, maintaining a balance between the antioxidants and free radicals [61]. A study showed that *Arabidopsis* overexpressing *FtNAC31* had weaker oxidative stress and increased the capability of the ROS-scavenging system [62]. In the present study, transgenic *Arabidopsis* plants overexpressing *SlNAC4* exhibited a higher accumulation of proline and betaine than control lines under salt and drought stress. Interestingly, we found that the photosynthetic efficiency of transgenic plants was superior to the WT and VT lines with increasing amounts of osmoregulatory substances under stress conditions, indicating that proline and betaine may maintain the photosynthetic activity of the plants by protecting the chloroplast (Figure 6A,B and Figure 7). This finding is similar to a previous study that three grapevine cultivars had a significant increase in proline content and the maximum photochemical quantum yield of photosystem II after cold stress [63]. Furthermore, the overexpression lines had significantly higher enzyme activities of SOD, POD, and CAT than those of the control and VT lines. Moreover, with the generation of oxidative damage, MDA (malondialdehyde) will accumulate to severely damage the cell membrane [64]. Concomitant with this increase in antioxidant enzyme activity in overexpression plants, we detected a reduction in the levels of MDA and electrolytic leakage, suggesting that transgenic *SlNAC4 Arabidopsis* plants may attenuate the membrane lipid peroxidation by improving the ROS-scavenging capacity, thus slowing down the oxidative damage caused by abiotic stress and enhancing tolerance to unfavorable survival conditions (Figure 6C–G). Considering these results, the overexpression of *SlNAC4* in transgenic *Arabidopsis* could improve the tolerance to salt and drought stresses.

The ABA signaling pathway is a central reaction pathway for environmental adversities. When plants are subjected to environmental stimuli, such as drought, salt, and extreme temperature, ABA accumulates to varying degrees [65]. Another study found that *OsAO3*-overexpressing *Nipponbare* has higher ABA content than that of wild-type plants under the drought treatment condition [66]. Our transgenic line’s data are similar (Figure 8A). Various ABA synthesis-related genes have been reported to be important for plant acclimation to environmental stress conditions. Heterologous expression of the *MsZEP* gene in *N. tabacum* could confer tolerance to drought and salt stress by affecting various physiological pathways, ABA levels, and stress-responsive genes expression [67]. Independent of its role in ABA biosynthesis, ABA3 enzyme regulates oxidative stress tolerance by regulating anthocyanin accumulation in *Arabidopsis* [68]. Research shows that *NCED3* is highly up-regulated in *HDA15*-overexpression transgenic plants, enhanced the accumulation of ABA, which promotes adaptation to salt stress [69]. Besides, it has been reported that AGL16 was able to bind the CArG motifs in the promoters of the *AAO3* and *CYP707A3*, leading to altered leaf stomatal density and ABA levels, hence acting as a negative regulator in drought resistance [70]. In the current study, we found that *Sl*NAC4 could significantly up-regulate the expression of several ABA synthesis-related genes, including *AtABA1*, *AtABA3*, *AtNCED3*, and *AtAAO3* (Figure 7). The ABA level in plants is regulated not only by its synthesis but also by decomposition. In soybean, the expression of *CYP707A3* genes were regulated by salt and drought stress in combination with ABA-related transcription factors, ultimately leading to changes in endogenous ABA levels [71]. *AtCYP707A3* was significantly lower in the overexpression plant under salt and drought stress (Figure 8B–D). Transcription factors can be involved in the regulation of multiple physiological activities by binding to downstream target genes. A yeast single-hybrid assay revealed that *Pp*NAC56, a prunus persica transcription factor, could bind to the promoter of *Pp*SnRK2D and *Pp*HSP17.4, leading to enhanced heat resistance in transgenic tomato [72]. In maize, a NAC transcription factor named *Zm*SNAC13 could activate or inhibit the expression of stress-responsive genes (*DREB*, *NF-YB3*, *YPL9*, *MPK3*, *WRKY53*, *Fes1A*), thereby enhancing the drought tolerance of transgenic *Arabidopsis* [73]. The yeast one-hybrid assay showed that *Sl*NAC4 could be combined with the promoters of *AtABA1*, *AtABA3*, *AtNCED3*, *AtAAO3*, and *AtCYP707A3* to activate or inhibit their expression, indicating that *Sl*NAC4 can participate in the ABA regulatory pathway through regulating the expression of ABA metabolism-related genes to enhance the adaptability of plants to a severe living environment (Figure 9).

## 4. Materials and Methods

### 4.1. Plant Materials and Growth Conditions

Green leaves and seeds of *Suaeda liaotungensis* were collected from Yingchengzi town of Dalian city in Liaoning Province. The leaves were quick-frozen with liquid nitrogen and stored in a freezer at −80 °C and used for genetic cloning. The seeds were stored at room temperature and germinated seeds were used for analysis of the expression characteristics of *SlNAC4*.

*Arabidopsis thaliana* (Columbia ecotype) was used for gene transformation and phenotypic analysis. After sowing in Murashige and Skoog (MS) solid medium and vernalizing for 3 days, culture dishes were transferred to an incubator (at 22 °C, with 16 h light/8 h darkness, a light intensity of 250 μmol·m^−2^·s^−1^) until the seedlings produced four true leaves. Seedlings were moved into potted soil and incubated in a growth chamber with 16 h light/8 h darkness, at 60% relative humidity and 22 °C.

### 4.2. Cloning and Bioinformatic Analysis of SlNAC4

Total RNA was isolated from leaves of *S. liaotungensis* using RNAiso Plus (TaKaRa), first-strand cDNA was synthesized using the PrimeScript^®^ RT Master Mix Perfect Real Time (Takara). The forward and reverse primers (EST-F and EST-R) (Table S1) used for *SlNAC4* cDNA amplification were designed based on the EST sequence of *SlNAC4*. The primers (SlNAC4-3′-Outer and SlNAC4-3′-Inner) (Table S1) were designed according to the confirmed EST sequences and the 3′ end fragment of the gene was amplified by 3’-Full RACE Core Set Ver.2.0 (TaKaRa). The EST sequence and 3’ end sequence were then spliced to obtain the *SlNAC4* sequence containing the complete coding region. The full-length *SlNAC4* fragment was obtained by PCR using the primer pair (SlNAC4-F and SlNAC4-R) (Table S1), and was thereafter cloned into a pEASY-T1 vector (TransGen Biotech, Beijing, China).

Sequence similarity and homology of *Sl*NAC4 were identified by performing Blast searches of the NCBI database (http://www.ncbi.nlm.nih.gov, accessed on 13 March 2021). The conserved domains in *Sl*NAC4 were analyzed using conserved domain finder software. Multiple sequence alignment was performed using GeneDoc. The NJ method was subsequently used to construct the phylogenetic trees.

### 4.3. Expression Analysis of SlNAC4 in Response to Different Stresses and ABA

*S. liaotungensis* seedlings with 6–8 true leaves were cultured in 1/10 Murashige and Skoog (MS) liquid medium for one week, and then root, stem, and leaf samples were collected for *SlNAC4* tissue-specificity expression analysis. Other seedlings were treated in 1/10 MS liquid medium supplemented with various concentrations of NaCl (200 mM), polyethylene glycol 20%, and ABA (0.1 mM), respectively. Leaves (0.1 g) were removed from the seedlings at 0, 0.5, 1, 3, 6, 12, and 24 h after different stresses treatment for total RNA extraction with RNAiso Plus (TaKaRa).

After the RNA extraction, cDNA was synthesized using a PrimeScript^®^ RT reagent kit with gDNA Eraser (Perfect Real Time) (TaKaRa). The transcription profile of *SlNAC4* was examined using quantitative real-time (qRT)-PCR with primer pair (SlNAC4-qF and SlNAC4-qR) (Table S1) and a SYBR^®^Premix Ex Taq™ (Perfect Real Time) kit (TaRaKa) in a Thermal Cycler Dice Real Time System TP800 (TaKaRa). *SlActin* (GenBank No. JX860282.1), used as the internal reference, was amplified with the primer pair (SlActin-qF and SlActin-qR) (Table S1). The 2^−ΔΔCT^ method was used to calculate relative gene expression and all experiments were repeated 3 times.

### 4.4. Subcellular Localization Analysis of SlNAC4

The ORF of *SlNAC4* was constructed on a pEGAD vector, which was controlled by CaMV 35S promoter to generate a 35S:*Sl*NAC4-GFP construct. Then the 35S:GFP and 35S:*Sl*NAC4-GFP recombinant vector were transferred into onion epidermal cells by biolistic bombardment using a PDS–1000/He™ Biolistic Particle Delivery System (Bio-Rad, Hercules, CA, USA). The Nikon Ti-E laser scanning confocal microscopy (Japan) was used to determine GFP fluorescence.

### 4.5. Transactivation Activity Analysis of SlNAC4

Full length or N-/C- terminal fragments of *SlNAC4* ORF were separately cloned into the GAL4 DNA-binding domain of the pGBKT7 vector to produce three recombinant vectors. The pGBKT7-*SlNAC4*-F, pGBKT7-*SlNAC4*-N, and pGBKT7-*SlNAC4*-C vectors and the control pGBKT7 vector were transferred into the yeast strain AH109, respectively. The transformants were cultured at 30 °C on SD/-Trp, SD/-Trp-His-Ade, and X-Gal medium for 3 days. After incubating, yeast growth status was observed and *β*-galactosidase activity was assessed.

### 4.6. Generation of Transgenic Arabidopsis Lines

To generate transgenic *Arabidopsis*, the *SlNAC4* ORF was cloned into pCAMBIA1300 the vector, which was regulated by the CaMV 35S promoter. The empty vector pCAMBIA1300 and recombinant vector pCAMBIA1300-*SlNAC4* were transformed into *Arabidopsis*, respectively, through *Agrobacterium*-mediated infiltration. Empty vector transgenic homozygous lines VT1 and VT2, the *SlNAC4* transgenic homozygous lines L1 and L2 with high *SlNAC4* expression level were selected through hygromycin resistance screening, PCR, and RT-PCR identification.

### 4.7. Seedling Growth Assays of SlNAC4 Transgenic Arabidopsis under Salt and Drought Stress

Wild-type *Arabidopsis* (WT), empty vector transgenic *Arabidopsis* lines (VT1,VT2), and *SlNAC4* transgenic lines (L1,L2) were used for phenotype analysis. For the seed germination experiment, the seeds of various lines were grown on MS solid medium (normal culture condition) and MS solid medium separately supplemented with different concentration of NaCl (0, 150, 200 mM) and D-mannitol (0, 200, 400 mM). Sixty seeds were sown for each line. The germination number of each line under normal culture and stress treatment was counted every 24 h and the exposure of radicle was used as the germination criterion. For the root elongation and lateral root growth experiment, the seeds were germinated on common MS medium, under salt stress (150 mM) or drought stress (200 mM) for 15 days. The root length and lateral root number of 30 seeds were calculated for each line. For survival ratio and growth status experiment, WT, VT1, VT2, L1, and L2 plants with four true leaves were transferred into a mixture of soil and vermiculite (1:1) in the sterile room for a week and treated without watering for drought stress or with 200 mM NaCl solution once every four days for salt stress. The survival ratio and growth status of each line under abiotic stress was recorded every seven days.

### 4.8. Measurement of Physiological Indicators of SlNAC4 Transgenic Arabidopsis

Under salt (200 mM NaCl) and drought (without watering for 21 days) treatments, WT, VT1, VT2, and transgenic plants with the same growth state were used to analyze physiological changes. The standard curve is drawn by measuring the absorbance with a spectrophotometer (Genova 2423, Bibby Scientific Ltd., Dunmow, Essex, UK) to measure the content of betaine and proline. The activities of the antioxidant enzymes such as superoxide dismutase (SOD), peroxidase (POD), and catalase (CAT) were determined using previously described methods [74]. Thereafter, we determined the MDA concentration was determined using the thiobarbituric acid method [75]. In addition, conductivity value was measured with an electrical conductivity meter (DDS-11A, Shanghai Pengshun Technology Co., Ltd., Shanghai, China). Besides, the chlorophyll fluorescence parameters of *Arabidopsis* leaves under salt and drought stress were analyzed with a chlorophyll fluorescence meter (IMAGING-PAM, Walz GmbH, Bad Waldsee, Germany), and the photosynthetic efficiency was calculated as follows: Fv/Fm = (Fm − F_0_)/Fm.

### 4.9. Analysis of ABA Content and ABA Metabolism Related Genes Expression in SlNAC4 Transgenic Arabidopsis thaliana

Wild-type and overexpression line (L1) were used as experimental material to detect the content of ABA by the double antibody sandwich method using the ABA ELISA Kit (RENJIEBIO, Shanghai, China) under salt and drought stress.

When the wild type and transgenic *Arabidopsis* grew to about 12 leaves, the seedlings were transferred to a one-half MS liquid medium for three days, after which they were treated for 6 h in one-half MS liquid medium, one-half liquid media containing 200 mM NaCl, and one-half MS liquid medium containing 10% PEG, respectively. In order to analyze the expression of ABA metabolism-related genes (*AtABA1, AtABA3, AtNCED3, AtAAO3*, and *AtCYP707A3*), total RNA was extracted from different *Arabidopsis* lines using RNAiso Plus (TaKaRa). The PrimeScript^®^ RT reagent kit with gDNA Eraser (Perfect Real Time) (TaKaRa) was used to synthesize the first-strand cDNA. The transcription of five genes was determined by qRT-PCR using the 2^−ΔΔCT^ method. All primer pairs (AtABA1-qF, AtABA1-qR, AtABA3-qF, AtABA3-qR, AtNCED3-qF, AtNCED3-qR, AtAAO3-qF, AtAAO-qR, AtCYP707A3-qF, AtCYP707A3-qR) for qRT-PCR analysis are listed in Table S1. Each experiment was performed with three independent biological replicates and three technical replicates.

### 4.10. Yeast One-Hybrid (Y1H) Assay

To identify whether the *Sl*NAC4 transcription factor can directly bind to *AtABA1*, *AtABA3*, *AtNCED3*, *AtAAO3* and *AtCYP707A3* gene promoters, the promoter core regions of the above five genes were amplified using primers (AtABA1-F, AtABA1-R, AtABA3-F, AtABA3-R, AtNCED3-F, AtNCED3-R, AtAAO3-F, AtAAO-R, AtCYP707A3-F, AtCYP707A3-R) (Table S1) and inserted into pAbAi vector to construct different bait vectors. And then the bait vectors were transferred into yeast strains Y1HGold and the bait yeast strains were obtained. The bait yeast strains were cultured on SD/-Ura media at 30 °C to assay their AbA background expression levels.

Similarly, the *SlNAC4* gene was inserted into pGADT7 using primers (SlNAC4-F, SlNAC4-R) (Table S1) to construct a pGADT7-*SlNAC4* vector. The prey vector (pGADT7-*SlNAC4*) was transformed into the bait yeast strains using the Preparation and Transformation Kit (Coolaber, Beijing, China) according to the manufacturer’s instructions. The recombinant yeast cells were restreaked on SD/-Leu medium with Aureobasidin A (0, 300, 800 ng/mL) for four days in a 30 °C incubator, and then observed for yeast growth.

### 4.11. Statistical Analysis

The data were analyzed using ANOVA in SPSS 21.0 software (SPSS, Inc., Chicago, IL, USA). A *p*-value < 0.05 or 0.01 was regarded as significant. Each experiment was performed in triplicate (transcriptional analysis *n* = 3, seed germination, primary root length, lateral root number, and survival analysis *n* = 30, physiological analysis *n* = 6).

## 5. Conclusions

**Figure 10:**
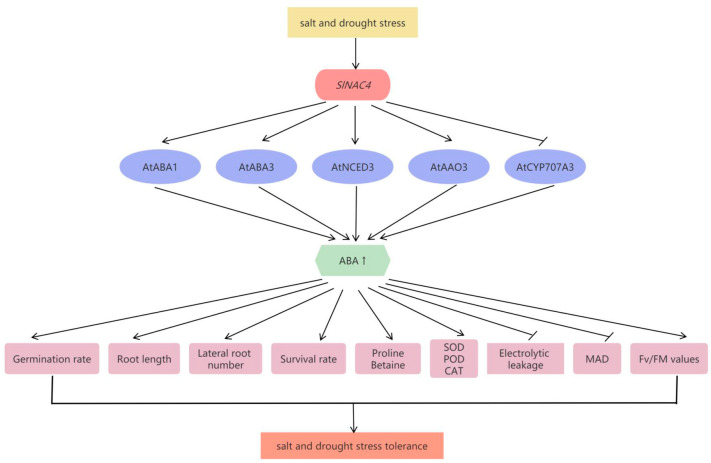
A proposed model for the regulatory function of *SlNAC4* in response to salt and drought stress. The expression level of *SlNAC4* is induced under salt and drought stress. *Sl*NAC4 can directly bind to the promoters of ABA metabolism-related genes (*AtABA1*, *AtABA3*, *AtNCED3*, *AtAAO3*, and *AtCYP707A3*) and activates or inhibits their expression. Increased ABA content alters the morphological features and enhances the physiological indicators of transgenic *Arabidopsis*, which can confer salt and drought tolerance to the plants.

In this study, a typical NAC gene *SlNAC4* was identified as a transcription activator from *S. liaotungensis* and functionally characterized in transgenic *Arabidopsis*. *SlNAC4* overexpression lines exhibited significantly enhanced salt and drought tolerance, and altered ROS-scavenging ability by regulating antioxidant enzyme activity in *Arabidopsis*. Furthermore, we observed that *SlNAC4*-overexpression transgenic plants had higher ABA content and *Sl*NAC4 can interact with numerous ABA metabolism related genes’ promoters and activate the transcription of their downstream genes. Taken together, our study illustrates that the transcription factor *Sl*NAC4 might participate in the salt and drought stress response via the ABA-mediated pathway in *Arabidopsis*. As such, we believe that *Sl*NAC4 can provide some new insight into the fundamental understanding of NAC transcription factors in improving plant adaption (Figure 10).

## Data Availability

Not applicable.

## Supplementary Materials

The following supporting information can be downloaded at: https://www.mdpi.com/article/10.3390/plants12162951/s1, Figure S1: Screening and identificating of *SlNAC4* transgenic *Arabidopsis*. Table S1: The primers sequences used for *SlNAC4* study.
