# Supplementary material for: A Transcription Factor SlNAC4 Gene of Suaeda liaotungensis Enhances Salt and Drought Tolerance through Regulating ABA Synthesis
Source: Plants (Basel). 2023 Aug 15;12(16):2951. doi: 10.3390/plants12162951 (PMC10459557; doi:10.3390/plants12162951)
Supplement: Supplementary file 1 [file plants-12-02951-s001.zip › Table S1.pdf]

Table S1. The primers sequences used for *SlNAC4* study.

Primer name	Primer sequence
EST-F	5’-CGAGTTTCAGCATCTGGGTT-3’
EST-R	5’-GAGCACGCCCTTG ATAGA-3’
SlNAC4-3’-Outer	5’-GGTGAATAAGTGGGGG GGAT-3’
SlNAC4-3’-Inner	5’-GAAAAGGAAGGTTGAAGGGC-3’
SlNAC4-F	5’-CATTCTGTGGGGGTTATTTG-3’
SlNAC4-R	5’-CCCTTACAAAGTTACAATGG-3’
SlNAC4-qF	5’-GAAAAGAAAGGTTGAAGGGCTAGA-3’
SlNAC4-qR	5’-ATCCCTTGGACAGAGAAAGAACC-3’
SlActin-qF	5’-ATCCCAAGGCTAA TCGTGAAAA-3’
SlActin-qr	5’-CACCATCACCAGAGTCCAACA-3’
AtABA1-qF	5’-ACTTAGGTGTTGGGCTTGGT-3’
AtABA1-qR	5’-CGGCTTTGTCAGTGAGTCTG-3’
AtABA3-qF	5’-CGTCGTCAGTGGAAGGTTTC-3’
AtABA3-qR	5’-AATTTCACCGGTCAGACCCT-3’
AtNCED3-qF	5’-TCTCCGGTGGTTTACGACAA-3’
AtNCED3-qR	5’-GGACCCTATCACGACGACTT-3’
AtAAO3-qF	5’-ACAAGGCACTTGGGACTACA-3’
AtAAO3-qR	5’-GGAATGTTTCCGAGCTTCCC-3’
AtCYP707A3-qF	5’-AAAGCCATGAAAGCTCGCAA-3’
AtCYP707A3-qR	5’-CTTGCGGCGAAGATTACTCC-3’
Atactin-qF	5’-CGAGGCTCCTCTTAACCCAA-3’
Atactin-qR	5’-ACCATCACCAGAATCCAGCA-3’
AtABA1-F	5’-cgagctcGCTCTAGCCTCTAGGCTATGGGT
AtABA1-R	5’-ccctcgagGTGACGTGATCAGCGCTTGTG
AtABA3-F	5’-ggggtaccCAAACCCGAATAGGTATCCAAAC
AtABA3-R	5’-ccctcgagACAATTTTCGTTTTCCACGTAAGT
AtNCED3-F	5’-ggggtaccACATATCCAAAAGTGACGATGA
AtNCED3-R	5’-gcgtcgacACCGTTGTAAGATGAAGTTAAGTGTA
AtAAO3-F	5’-cgagctcATGTGAAAGCTGTAGACTCGTTCG

AtAAO3-R	5'-ccctcgagTCATTGACTGTGCGCCACGAGT
AtCYP707A3-F	5'-ggggtaccAGTCTTTGGTCCTAGATCCCTCAAG
AtCYP707A3-R	5'-ccctcgagAGGCAGTGGAGGAGAGAGAAG
SlNAC4-F	5'-cggaattcATGGGAGCTGCAACTTTG
SlNAC4-R	5'-ccctcgagTCAGAAGGGATTTTGATAGCTAAGAGTA
