# Supplementary figures and images for: A Transcription Factor SlNAC4 Gene of Suaeda liaotungensis Enhances Salt and Drought Tolerance through Regulating ABA Synthesis
Source: Plants (Basel). 2023 Aug 15;12(16):2951. doi: 10.3390/plants12162951 (PMC10459557; doi:10.3390/plants12162951)

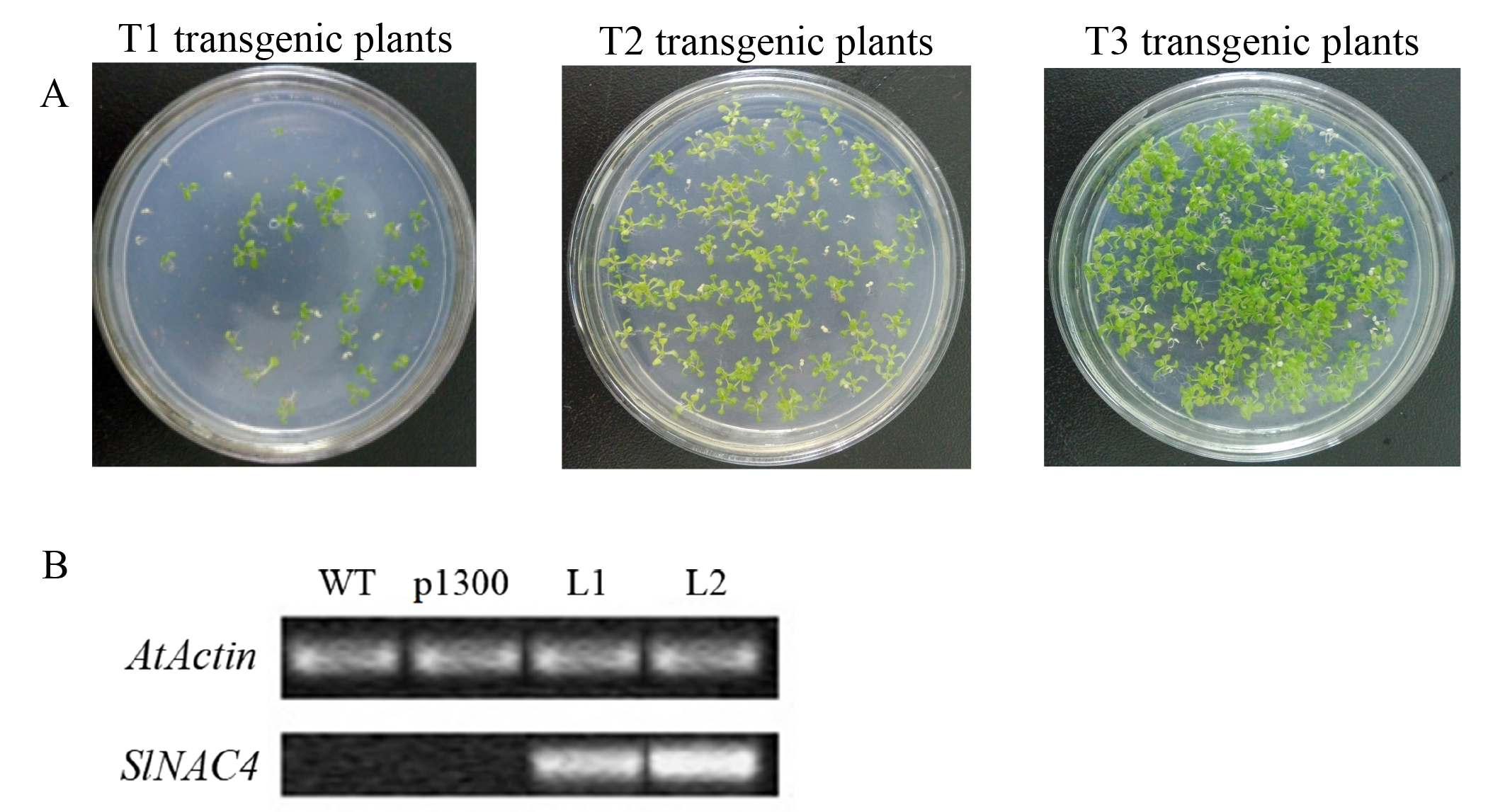

Supplement: Supplementary file 1 [file plants-12-02951-s001.zip › Figure S1.tif]
